# Gender Disparities in COVID-19 Survivors with Impaired Quality of Life, Effort Intolerance, and Cardiopulmonary Symptoms: A Prospective Cohort Study

**DOI:** 10.1089/whr.2024.0131

**Published:** 2025-01-28

**Authors:** Krista Zachariah, Dustin Wessells, Prianca Tawde, Mahniz Reza, Caitlin Chiu, Pablo Villar Calle, Alexander Volodarskiy, Evelyn M. Horn, Parag Goyal, Jonathan Weinsaft, Jiwon Kim

**Affiliations:** ^1^Department of Medicine (Cardiology), Weill Cornell Medicine, New York, New York, USA.; ^2^Department of Medicine (Cardiology), New York, Presbyterian Queens, Flushing, New York, USA.

**Keywords:** cardiac MRI, cardiopulmonary symptoms, COVID-19, echocardiogram, effort tolerance, quality of life

## Abstract

**Background:**

Prior studies have suggested gender differences in COVID-related outcomes that have the potential to impact cardiovascular risk. We aimed to investigate gender differences on short- and long-term effects of COVID-19 infection.

**Methods:**

Patients hospitalized with COVID-19 infection were enrolled in an ongoing prospective registry across NY-Presbyterian networks, which encompassed same-day echocardiogram, cardiac magnetic resonance (CMR), 6-minute walk test, and quality of life assessment 1 year following acute COVID hospitalization.

**Results:**

In this prospective cohort of 213 hospitalized patients with COVID-19 infection, males were more likely to require intensive care unit (ICU) stay (13.6 vs. 3.6%; *p* = 0.009) and oxygen supplementation (40.8 vs. 26.4%; *p* = 0.026), paralleling higher rates of elevated troponin, C-reactive protein, ferritin, and D-dimer (*p* < 0.05 for all). In contrast, 1 year following COVID hospitalization, females reported worse physical function and fatigue on Patient-Reported Outcomes Measurement Information System (PROMIS) scale (*p* < 0.05 for all). Additionally, 6-minute walk distance was less in females than males (383.0 ± 98.0 vs. 428.6 ± 78.6 m; *p* = 0.006), and Borg dyspnea score was nearly twofold higher in females vs. males (2.0 ± 2.3 vs. 1.0 ± 1.5; *p* < 0.001). With respect to imaging parameters, females had higher left ventricle and right ventricle ejection fraction (*p* < 0.05 for all) with smaller infarct size (*p* = 0.042) on CMR.

**Conclusion:**

Whereas males have greater morbidity during acute COVID hospitalization, females are disproportionately impacted by post-COVID impaired functional status despite higher biventricular ejection fraction and smaller infarct size.

## Introduction

Coronavirus disease 2019 (COVID-19) is an ongoing global pandemic that has affected more than 103 million people in the United States.^1^ Despite the substantial mortality risk, a growing population of COVID-19 patients survive acute illness but remain at risk for long-term deleterious consequences, including impaired exercise tolerance.^2^ Of patients surviving from COVID, a significant proportion has persistent symptoms or new symptoms attributed to their initial infection.^3^ In 2021, the diagnosis of “long COVID” was defined as the persistence of physical and/or psychological symptoms for more than four to 12 weeks after recovery from acute COVID-19 disease.^4^ Many patients experiencing long COVID specifically report cardiopulmonary symptoms, such as dyspnea, chest pain, and palpitations.^5^ While many studies have looked at the cardiovascular implications of COVID-19 in the acute and long-term phases, there is still more understanding needed of the cardiovascular outcomes related to COVID-19 infection.

Prior studies have shown that there are significant differences in both short- and long-term complications of COVID when stratifying by gender, which can potentially impact cardiovascular risk. The results show that women tend to experience less severe acute infections but suffer from long COVID more frequently.^6–9^ The underlying mechanism for this gender disparity is currently unknown. In this study, we aim to investigate these gender differences in COVID-19 outcomes using a prospective cardiovascular imaging study, which evaluates both baseline and follow-up characteristics of COVID-19 survivors to better understand whether there are cardiovascular structural differences that may be attributable to observed gender differences.

## Methods

### Data source and study population

A total of 213 COVID-19 survivors were recruited through ongoing registries of COVID-19 patients in the NewYork-Presbyterian Hospital networks and were cross-referenced with electronic medical records to screen for inclusion and exclusion criteria. Patients were then contacted for follow-up, and multiple attempts were made to ensure a representative cohort, regardless of the severity of long-term symptoms. Men and women aged ≥18 years with documented COVID-19 infection, inclusive of patients with residual cardiopulmonary symptoms and asymptomatic patients, were included. Patients who had a contraindication to cardiac magnetic resonance imaging (MRI), an inability to provide informed consent, and/or an unrelated condition with life expectancy <12 months were excluded. All patients provided informed written consent at the time of enrollment. This study was conducted with the approval of the institutional review board at Weill Cornell Medicine.

### Study variables and end points

Baseline data were collected to assess demographics, initial disease severity, including biomarkers of inflammation/thrombosis and cardiac structure/function, treatments received, and inpatient complications. Gender was defined as male vs. female based upon sex assigned at birth in the electronic medical record. Biomarker data encompassed prespecified indices generally associated with adverse prognosis, including troponin, B-type natriuretic peptide, C-reactive protein, erythrocyte sedimentation rate, ferritin, D-dimer, white blood cell count, hemoglobin, hematocrit, and platelet count. For patients with biomarkers obtained at multiple time points, peak values were used for study-related data analyses. Elevated thresholds for elevated biomarkers were defined based on site-specific laboratory thresholds at participatory hospitals. Intensive care unit (ICU) admission included any ICU stay during hospitalization. Hypoxia was defined as any need for supplemental oxygenation. Acute kidney injury was defined as an increase in serum creatinine by 0.3 mg/dL (26.5 mol/L) within 48 hours or an increase in serum creatinine to 1.5 baseline within the prior 7 days. Vasopressor use was defined as need for any vasopressor support during hospitalization.

After infection, patients were evaluated by study team members to assess myocardial tissue properties, functional recovery, effort tolerance, and short-term symptoms after COVID-19 infection. Post-acute sequelae of COVID-19 (PASC) were assessed using a symptom-limited 6-minute walk test (100-foot corridor). Clinical symptoms were assessed using surveys including the Patient-Reported Outcomes Measurement Information System (PROMIS-29 Questionnaire), which consist of a group of patient-reported outcome measures that span a wide array of physical, social, and emotional health outcomes. Patient dyspnea was evaluated utilizing the Borg dyspnea score. Myocardial tissue properties were assessed *via* cardiac MRI and echocardiography. Cardiac MRI was performed to assess structural/functional remodeling, myocardial tissue properties (fibrosis and edema), and cardiopulmonary blood oxygenation. Magnetic resonance angiography was acquired adjunctively during IV gadolinium administration. Echocardiogram was performed using commercial transthoracic ultrasound equipment.

### Statistical analysis

All analyses were performed using IBM SPSS Statistics version 27.0 (SPSS, Chicago, IL). Continuous variables are summarized as means ± standard deviations. Categorical variables are summarized as frequencies and percentages. Normally distributed continuous indices were compared *via t*-tests (for two group comparisons) or analysis of variances with linear test of trends (for multiple group comparisons). Categorical variables were compared using chi-square tests. A two-sided *p* < 0.05 was considered statistically significant.

## Results

In this prospective cohort of COVID-19 survivors, there were significant differences observed in acute hospitalization variables when stratified by gender (Table 1). Male patients had 3.5-fold longer ICU stays when compared with female patients (*p* = 0.009). Male patients had higher rates of hypoxia (*p* = 0.026) and were more likely to require nasal cannula use (*p* = 0.023). There were higher rates of acute kidney injury in males at 14.6% compared with 3.6% of females (*p* = 0.005). There were no differences in cardiac arrest, vasopressor use, or COVID-19 therapy use. For the lab analyses done during the hospitalization, males had higher rates of troponin elevations (*p* = 0.018), higher hemoglobin (*p* < 0.001), higher hematocrit (*p* < 0.001), higher ferritin (*p* = 0.013), and higher D-dimer (*p* = 0.033).

**Table 1.** Acute COVID Hospitalization of COVID-19 Survivors with Cardiac Sequalae Stratified by Gender

	All (*n* = 213)	Male (*n* = 103)	Female (*n* = 110)	Odds ratio^a^	*p*
Hospitalization characteristics					
ICU stay	18 (8.5)	14 (13.6)	4 (3.6)	4.2	**0.009**
ICU length of stay (days)	5.3 ± 14.7	8.1 ± 20.1	2.7 ± 5.1		**0.009**
Oxygenation support	71 (33.3)	42 (40.8)	29 (26.4)	1.9	**0.026**
Nasal cannula use	61 (28.6)	37 (35.9)	24 (21.8)	2.0	**0.023**
Mechanical ventilation	9 (4.2)	7 (6.8)	2 (1.8)	3.9	0.093
Acute kidney injury	19 (8.9)	15 (14.6)	4 (3.6)	4.5	**0.005**
Cardiac arrest	1 (0.5)	1 (1.0)	0 (0.0)	0.5	0.484
Vasopressor use	5 (2.3)	3 (2.9)	2 (1.8)	1.6	0.675
Steroid use	72 (33.8)	37 (35.9)	35 (31.8)	1.2	0.527
Remdesivir	52 (24.4)	26 (25.2)	26 (23.6)	1.1	0.785
Tocilizumab	7 (3.3)	6 (5.8)	1 (0.9)	6.7	0.058
Paxlovid	21 (9.9)	9 (8.7)	12 (10.9)	0.8	0.595
Plaquenil	28 (13.1)	17 (16.5)	11 (10.0)	1.8	0.160
Monoclonal antibodies	14 (6.6)	6 (5.8)	8 (7.3)	0.8	0.670
Readmission	27 (12.7)	11 (10.7)	16 (14.5)	0.7	0.397
Vaccination	201 (94.4)	98 (95.1)	103 (93.6)	1.3	0.633
Laboratory values					
Troponin (ng/L)	0.5 ± 2.8	0.9 ± 3.7	0.1 ± 0.3		0.123
ULN troponin, *n* = 131	17 (13.0)	13 (20.0)	4 (6.1)	3.9	**0.018**
BNP (pg/mL)	137.7 ± 450.3	149.1 ± 503.0	121.9 ± 372.5		0.800
ULN BNP, *n* = 89	12 (13.5)	7 (14.9)	5 (11.9)	1.3	0.680
CRP (mg/dL)	14.2 ± 33.8	14.7 ± 15.7	13.4 ± 48.7		0.859
ULN CRP, *n* = 109	86 (78.9)	52 (88.1)	34 (68.0)	3.5	**0.010**
ESR (mm/hour)	58.4 ± 36.3	61.4 ± 40.4	54.3 ± 30.3		0.373
ULN ESR, *n* = 85	66 (77.6)	38 (82.6)	28 (71.8)	1.9	0.233
WBC (10^3^/uL)	8.9 ± 5.5	10.1 ± 5.8	7.8 ± 5.0		**0.006**
ULN WBC, *n* = 181	61 (33.7)	35 (38.9)	26 (28.6)	1.6	0.142
Platelet (10^3^/uL)	281.0 ± 138.0	290.1 ± 151.1	272.0 ± 123.9		0.379
ULN platelet, *n* = 180	37 (20.6)	22 (24.4)	15 (16.7)	1.6	0.197
Hematocrit (%)	40.1 ± 5.7	41.8 ± 4.9	38.4 ± 6.0		**<0.001**
ULN hematocrit, *n* = 179	57 (31.8)	39 (43.8)	18 (20.0)	3.1	**<0.001**
Ferritin (ng/mL)	773.5 ± 905.9	972.7 ± 942.0	526.6 ± 801.9		**0.011**
ULN ferritin, *n* = 90	61 (67.8)	38 (79.2)	23 (54.8)	3.1	**0.013**
D-dimer (ng/L)	1185.7 ± 2771.1	1815.7 ± 3569.8	370.5 ± 313.4		**0.011**
ULN D-dimer, *n* = 108	58 (53.7)	34 (64.2)	24 (43.6)	2.3	**0.033**
LDH (U/L)	392.3 ± 449.7	442.5 ± 555.8	318.4 ± 200.1		0.203
ULN LDH, *n* = 82	68 (82.9)	43 (89.6)	25 (73.5)	3.1	0.057
Hemoglobin (g/dL)	13.3 ± 2.0	14.0 ± 1.7	12.5 ± 2.1		**<0.001**
ULN hemoglobin, *n* = 180	57 (31.7)	39 (43.3)	18 (20.0)	3.2	**<0.001**
Symptom status					
Chest pain	62 (29.1)	28 (27.2)	34 (30.9)	0.8	0.550
Shortness of breath	54 (25.4)	21 (20.4)	33 (30.0)	0.6	0.107
Fatigue	84 (39.4)	26 (25.2)	58 (52.7)	0.3	**<0.001**

Bolded data in tables refers to statistically significant P-values.

^a^ Exposure is assigned as female gender.

BNP, brain natriuretic peptide; CRP, C-reactive protein; ESR, erythrocyte sedimentation rate; ICU, intensive care unit; LDH, lactate dehydrogenase; ULN, upper limit of normal; WBC, white blood cell.

In the post-hospitalization follow-up with the PROMIS scale (Table 2), females reported lower physical function (*p* = 0.004) and social participation (*p* = 0.001). Females were more likely to have post-COVID fatigue (*p* < 0.001), depression (*p* = 0.011), and anxiety (*p* = 0.005). No significant differences were observed in subgroup analysis of women stratified by age (Table 3). Table 4 demonstrates that males walked farther in the 6-minute walk test (428.6 ± 78.6 vs. 393.0 ± 98.0, *p* = 0.006). Females reported more dyspnea after the test in the Borg dyspnea score (2.0 ± 2.3 vs. 1.0 ± 1.5, *p* < 0.001) and a significantly higher difference in the pre and postdyspnea score (*p* < 0.001). Females reported higher Borg fatigue scores pre and post (*p* < 0.001), but there was no significant change in fatigue between the two groups. In Table 5, post-infection cardiac imaging shows differences in myocardial structure and tissue substrate. For example, female patients had higher left ventricular ejection fractions by both echo (*p* = 0.002) and cardiac MRI (*p* = 0.001). Similarly, female patients also had higher right ventricular ejection fraction by cardiac MRI (*p* < 0.001). Regarding tissue characterization, females had smaller infarct size (*p* = 0.042) despite a similar prevalence of late gadolinium enhancement (LGE) by gender.

**Table 2.** PROMIS Scales in COVID-19 Survivors When Stratified by Gender

	All (*n* = 213)	Male (*n* = 103)	Female (*n* = 110)	*p*
Physical function	48.0 ± 9.5	49.9 ± 8.2	46.2 ± 10.3	**0.004**
Social participation	52.6 ± 10.6	55.1 ± 9.1	50.4 ± 11.4	**0.001**
Anxiety	52.2 ± 10.8	50.0 ± 10.0	54.2 ± 11.2	**0.005**
Depression	48.0 ± 9.1	46.5 ± 8.2	49.4 ± 9.7	**0.011**
Fatigue	49.7 ± 11.5	46.5 ± 10.3	52.8 ± 11.8	**<0.001**
Sleep disturbance	51.4 ± 5.2	50.6 ± 5.0	52.2 ± 5.3	**0.037**

**Table 3.** PROMIS Scales in COVID-19 Survivors When Stratified by Age in Women

	All (*n* = 110)	Under 50 (*n* = 50)	Over 50 (*n* = 60)	*p*
Physical function	46.2 ± 10.3	47.3 ± 10.0	45.3 ± 10.6	0.307
Social participation	50.4 ± 11.4	51.1 ± 11.7	49.8 ± 11.7	0.555
Anxiety	54.2 ± 11.2	55.3 ± 10.3	53.2 ± 11.9	0.327
Depression	49.4 ± 9.7	49.3 ± 9.6	49.5 ± 9.9	0.888
Fatigue	52.8 ± 11.8	54.9 ± 12.2	51.0 ± 11.3	0.082
Sleep disturbance	52.2 ± 5.3	51.4 ± 5.5	52.7 ± 5.2	0.223

**Table 4.** Six-Minute Walk Test and Dyspnea in COVID-19 Survivors When Stratified by Gender

	All (*n* = 213)	Male (*n* = 103)	Female (*n* = 110)	*p*
Heart rate pre (bpm)	73.7 ± 11.4	72.2 ± 12.2	75.0 ± 10.5	0.088
Heart rate post (bpm)	79.6 ± 13.7	78.2 ± 14.0	81.0 ± 13.4	0.174
Heart rate difference (bpm)	0.1 ± 2.4	0.0 ± 2.3	0.2 ± 2.5	0.532
Systolic BP pre (mmHg)	135.0 ± 18.9	137.1 ± 16.6	133.0 ± 20.6	0.140
Systolic BP post (mmHg)	135.7 ± 19.3	138.3 ± 16.7	133.4 ± 21.2	0.082
Systolic BP difference(mmHg)	0.8 ± 12.1	1.2 ± 11.8	0.4 ± 12.3	0.649
Diastolic BP pre (mmHg)	83.5 ± 11.4	84.8 ± 10.6	82.2 ± 12.0	0.125
Diastolic BP post (mmHg)	84.2 ± 11.5	85.5 ± 10.7	82.9 ± 12.2	0.121
Diastolic BP difference (mmHg)	0.1 ± 2.3	0.1 ± 2.6	0.2 ± 2.0	0.608
Time walked (mins)	5.9 ± 0.5	5.9 ± 0.5	5.9 ± 0.4	0.483
Predicted distance walked (m)^a^	569.6 ± 98.7	583.2 ± 103.3	556.8 ± 92.7	**0.050**
Distance walked (m)	410.0 ± 90.8	428.6 ± 78.6	393.0 ± 98.0	**0.006**
Proportion of predicted distance walked (m)	0.7 ± 0.2	0.7 ± 0.2	0.7 ± 0.2	0.424
Borg dyspnea pre	0.5 ± 1.1	0.3 ± 0.9	0.6 ± 1.3	0.094
Borg dyspnea post	1.5 ± 2.0	1.0 ± 1.5	2.0 ± 2.3	**<0.001**
Borg dyspnea difference	1.0 ± 1.6	0.6 ± 1.2	1.4 ± 1.7	**<0.001**
Borg fatigue pre	1.1 ± 1.7	0.6 ± 1.3	1.5 ± 2.0	**<0.001**
Borg fatigue post	1.5 ± 2.1	1.0 ± 1.6	2.0 ± 2.3	**0.001**
Borg fatigue difference	0.4 ± 1.6	0.4 ± 1.0	0.5 ± 2.0	0.717

^a^ Predicted distance walked was calculated using reference equations for 6-minute walk in healthy adults.^16^

BP, blood pressure.

**Table 5.** Imaging Characteristics in COVID-19 Survivors When Stratified by Gender

	All (*n* = 213)	Male (*n* = 103)	Female (*n* = 110)	*p*
Echo values				
LVEF	63.9 ± 6.9	62.2 ± 7.4	65.4 ± 6.3	**0.002**
LVIDd	4.8 ± 0.5	5.0 ± 0.5	4.6 ± 0.4	**<0.001**
LVIDs	3.1 ± 0.5	3.3 ± 0.5	2.9 ± 0.4	**<0.001**
LVEDV (indexed)	57.7 ± 11.9	58.9 ± 11.4	56.7 ± 12.3	0.235
LVESV (indexed)	21.2 ± 7.7	22.6 ± 7.7	20.0 ± 7.4	**0.018**
LV mass (indexed)	73.3 ± 19.0	81.3 ± 21.8	66.3 ± 12.5	**<0.001**
LA volume index	25.6 ± 10.1	26.5 ± 12.4	24.7 ± 7.2	0.223
RVDd	3.7 ± 0.9	4.1 ± 0.4	3.4 ± 1.2	**0.012**
PASP	26.3 ± 10.1	26.6 ± 5.8	26.0 ± 5.8	0.543
Cardiac MRI values				
LVEDV (indexed)	70.2 ± 15.1	72.6 ± 16.1	67.9 ± 13.7	**0.031**
LVESV (indexed)	26.3 ± 13.8	28.8 ± 16.8	23.9 ± 10.0	**0.014**
LVEF	64.3 ± 8.2	62.5 ± 8.3	66.0 ± 7.4	**0.001**
RVEDV (indexed)	75.5 ± 18.0	78.3 ± 17.9	73.0 ± 17.8	**0.047**
RVESV (indexed)	32.7 ± 11.5	35.0 ± 10.3	30.6 ± 12.2	**0.009**
RVEF	57.8 ± 6.3	56.1 ± 5.7	59.3 ± 6.5	**<0.001**
LV mass (indexed)	53.8 ± 12.6	59.3 ± 13.5	48.9 ± 9.5	**<0.001**
Prevalence of LGE	0.1 ± 0.3	0.2 ± 0.4	0.1 ± 0.3	0.090
Global LV infarct size^a^	0.6 ± 3.2	1.1 ± 4.5	0.2 ± 0.7	**0.042**

^a^ LV infarct size includes only patients with LGE.

LA, left atrium; LGE, late gadolinium enhancement; LV, left ventricle; LVEDV, left ventricular end-diastolic volume; LVEF, left ventricular ejection fraction; LVESV, left ventricular end-systolic volume; LVID, Left ventricular internal dimension; LVIDd, left ventricular internal dimension in diastole; LVIDs, left ventricular internal dimension in systole; MRI, magnetic resonance imaging; PASP, pulmonary artery systolic pressure; RVEDV, right ventricular end-diastolic volume; RVEF, right ventricular ejection fraction; RVESV, right ventricular end-systolic volume.

## Discussion

Our study shows that men have worse acute COVID-19 infections, including longer ICU stays, higher needs for supplemental oxygen, and higher rates of elevated inflammatory markers.

This hypothesis is in line with previous studies, which implicate a systemic inflammatory response to COVID causing structural remodeling of cardiac or pulmonary system through the fibrosis activation pathway.^10^ Our data showed inflammatory markers appeared to be lower in females as compared to men during acute infection, which appears to benefit females in the acute phase of infection. Beyond end-organ damage from acute phase infection, the cause of long COVID symptoms is still unclear. Other studies have highlighted this trend of worse acute infection in males and higher incidence of long COVID symptoms and mood disorders in women.^11^ However, immune dysregulation appears to play a role.^10^

Long COVID has also been characterized by impaired systemic oxygen extraction seen on invasive cardiopulmonary exercise testing. The spectrum of phenotypes of long COVID correlates to different degrees of impaired oxygen extraction associated with aberrant protein expression and cardiopulmonary physiological response. Patients with severely impaired oxygen extraction appear to show a maladaptive physiological and proteomic signature, which is consistent with persistent inflammatory state and endothelial dysfunction. The exertional intolerance appears to be related more to impaired oxygen extraction and is independent of cardiac etiologies, given that there are no long-term cardiopulmonary disease sequalae.^12^ Impaired peripheral oxygen extraction, rather than cardiac dysfunction, may therefore be another major factor contributing to this gender difference, which is further supported by our data showing favorable cardiac imaging parameters in female patients.

Another factor that may explain the gender difference in COVID-19 outcomes is the role of increased estrogen levels. A 2021 study found that women under 60 were more likely to develop long COVID, with the risk leveling off after age 60.^13^ This led to the hypothesis that long COVID could be an estrogen-mediated condition, given its higher prevalence in younger females compared to males. This hypothesis suggests that symptoms such as dyspnea and fatigue, as observed in our follow-up, could be related to estrogen’s influence on long COVID. Although our current study was not designed to test this hypothesis specifically, further research with larger populations is needed to validate these findings.

Finally, autonomic dysfunction has also been seen as a mediator of long COVID.^14^ The symptoms of long COVID have been recognized as similar to those of postural orthostatic tachycardia syndrome (POTS). POTS has been considered a major phenotype in the new postacute COVID-19 syndrome, with an approximate prevalence of 30% in highly symptomatic patients with long COVID.^14^ This finding appears to be unrelated to the severity of the initial infection. The mechanism of COVID-19 postinfection autonomic dysfunction is thought to be multifactorial. Given that acute COVID-19 infection and COVID-19 vaccination can trigger POTS and other types of cardiovascular autonomic dysfunction, it has been hypothesized that these factors might be potent immune triggers that evoke an autoimmune response in susceptible individuals. Microvascular dysfunction has also been hypothesized to be a factor contributing to autonomic dysfunction in COVID, thereby preferentially affecting young- and middle-aged women, possibly suggesting a mechanistic role for sex hormones.^14^ Other proposed mechanisms include persistent viremia leading to a persistently high inflammatory state with cellular injury, cytokine- and hypoxia-induced injury leading to neuronal apoptosis causing impaired neurological function, and the inflammatory pathway leading to autonomic and small fiber neuropathies, as previously seen in viral infections from herpes simplex and infectious mononucleosis.^15^ As more data arises, research can focus on phenotyping of autonomic dysfunction in cohorts of patients with long COVID syndrome to identify biomarkers of the condition and ultimately focus on effective therapies.

This study has several limitations, including the lack of stratification by menopausal status and the absence of long-term cardiovascular outcomes. While many results were statistically significant, the clinical relevance of these findings requires further investigation, particularly given the emerging role of various factors, as previously mentioned. Additionally, autonomic dysfunction has been proposed as a possible mediator of long COVID symptoms, but a limitation of our study is the absence of autonomic dysfunction measures. Although we observed higher ejection fractions and smaller infarct sizes in female patients, long-term cardiovascular outcomes were not the primary focus of this research. Future studies with larger cohorts and extended follow-up periods are needed to better understand the mechanisms driving these differences and to assess whether gender-related differences translate into meaningful long-term health outcomes. Moreover, research with longer follow-up durations will be crucial in addressing these important questions and further clarifying the interplay between sex hormones, immune responses, autonomic dysfunction, and impaired systemic oxygen extraction in shaping the long-term consequences of COVID-19 infection.

## Conclusion

This study demonstrates that males have greater morbidity during acute COVID hospitalization than female patients with higher rates of ICU admissions, hypoxia, acute kidney injury, and elevations in troponin and inflammatory markers. Female patients were disproportionately impacted by post-COVID symptoms of fatigue, anxiety, and depression. Female patients performed worse in the 6-minute walk test and reported more dyspnea and fatigue than males. Contrary to these symptoms, cardiac imaging parameters demonstrated significantly better biventricular ejection fractions in females with trend toward smaller infarct size. Findings of this study contribute to improved understanding of gender-specific impacts of COVID-19 and have the potential to inform targeted interventions toward the goal of mitigating gender-based cardiovascular risks associated with COVID-19 infection.

## Data Availability

The datasets used and/or analyzed during the current study are available from the corresponding author upon reasonable request.

## Abbreviations Used

BNP: brain natriuretic peptide
BP: blood pressure
COVID-19: coronavirus disease 2019
CMR: cardiac magnetic resonance
CRP: c-reactive protein
ESR: erythrocyte sedimentation rate
ICU: intensive care unit
LA: left atrium
LDH: lactate dehydrogenase
LGE: late gadolinium enhancement
LV: left ventricle
LVEDV: left ventricular end-diastolic volume
LVEF: left ventricular ejection fraction
LVESV: left ventricular end-systolic volume
LVIDd: left ventricular internal dimension in diastole
LVIDs: left ventricular internal dimension in systole
MRI: magnetic resonance imaging
PASP: pulmonary artery systolic pressure
PROMIS: Patient-Reported Outcomes Measurement Information System
POTS: postural orthostatic tachycardia syndrome
RA: right atrium
RV: right ventricle
RVEDV: right ventricular end-diastolic volume
RVEF: right ventricular ejection fraction
RVESV: right ventricular end-systolic volume
ULN: upper limit of normal
WBC: white blood cell
